# Shielding Properties of Some Marble Types: A Comprehensive Study of Experimental and XCOM Results

**DOI:** 10.3390/ma14154194

**Published:** 2021-07-27

**Authors:** Mohamed Elsafi, Mohamed A. El-Nahal, M. F. Alrashedi, O. I. Olarinoye, M. I. Sayyed, Mayeen Uddin Khandaker, Hamid Osman, Sultan Alamri, M. I. Abbas

**Affiliations:** 1 Physics Department, Faculty of Science, Alexandria University, Alexandria 21511, Egypt; matab.1413@gmail.com (M.F.A.); mabbas@physicist.net (M.I.A.); 2 Department of Environmental Studies, Institute of Graduate Studies and Research, Alexandria University, Alexandria 21526, Egypt; Igsr.nahalmoh@alexu.edu.eg; 3 Physics Department, College of Science and Arts, Al-Qassim University, Buraydah 52571, Saudi Arabia; 4 Department of Physics, Federal University of Technology, Minna 920271, Nigeria; leke.olarinoye@futminna.edu.ng; 5 Department of Physics, Faculty of Science, Isra University, Amman 11622, Jordan; dr.mabualssayed@gmail.com; 6 Department of Nuclear Medicine Research, Institute for Research and Medical Consultations, Imam Abdulrahman bin Faisal University, Dammam 31441, Saudi Arabia; 7 Centre for Applied Physics and Radiation Technologies, School of Engineering and Technology, Sunway University, Bandar Sunway 47500, Selangor, Malaysia; mayeenk@sunway.edu.my; 8 Department of Radiological Sciences, College of Applied Medical Sciences, Taif University, Taif 21944, Saudi Arabia; ha.osman@tu.edu.sa (H.O.); s.alamri@tu.edu.sa (S.A.)

**Keywords:** marbles, mass attenuation coefficient, half value layer, buildup factor, fast neutron removal cross section

## Abstract

In this work, some marble types were collected from Egypt, and their shielding characteristics were estimated. Their rigidity, in addition to their elegant shape, led us to consider their use as a protective shield, in addition to making the workplace more beautiful. The mass attenuation coefficient (*μ*/*ρ*) was calculated for three types of marble (Breshia, Galala, and Trista) experimentally, using a narrow gamma ray source and high pure germanium (HPGe). The results obtained were compared with the XCOM program and indicated a very good agreement between the two methods. The linear attenuation coefficient (*μ*) was evaluated to calculate the half and tenth value layers. The maximum *μ* value of 1.055, 1.041, and 1.024 cm^−1^ was obtained for Breshia, Galala, and Trista, respectively, at 0.06 MeV. The mean free path for studying the materials was compared with other shielding materials and showed good results at different energy scales. The energy absorption (EABF) and exposure buildup factors (EBF) were determined at different mean free paths. The fast neutron removal cross section Σ_R_ was calculated and expresses the ability of the marbles to slow down fast neutrons through multiple scattering. This is the ability of the marbles to shield fast neutrons.

## 1. Introduction

With the increased use of radiation across vast fields of work, it is necessary to also develop radiation shields that can adequately protect the bodies of workers and patients that come in contact with high energy photons. Radiation shields, or materials that are used to absorb radiation, are necessary to properly attenuate ionizing radiation, which is defined as radiation that has sufficient energy to detach electrons from atoms. Long-term human exposure to ionizing radiation can cause permanent tissue damage, acute radiation syndrome, cancer, and death in extreme cases. Thus, to prevent these effects, workers and patients must be provided with efficient radiation shields that will lower the levels of radiation to safe enough levels [1,2,3,4].

When deciding on a material to use to attenuate radiation, several specifics of the application must be considered, such as the energies of the incoming photons, the environmental conditions of the radiation source (indoors or outdoors), whether transparency is necessary, etc. Due to these varying uses, a shield that may be ideal for one specific situation may not be an effective shield in another. Some examples of commonly used radiation shielding materials include concrete, alloys, pure lead, and glasses. All these materials offer their own unique pros and cons but are receiving attention from various researchers in the radiation shielding community to attempt to discover the ideal shielding material for each application [5,6,7,8,9,10].

As a rule of thumb, dense materials with high atomic numbers offer the best attenuation capability, as density typically correlates with a better shielding ability. Lead, iron, and materials that contain these two elements are examples of dense radiation shields. Although these materials offer desirable shielding properties, pure lead and iron cannot be used in the construction of buildings because of their durability and their high cost. Additionally, lead is toxic to humans and the environment, which makes it less than ideal as a radiation shield, and especially as a building material. Therefore, less effective, but cheaper shielding materials are mostly used, such as sand, bricks, cement, and concrete. However, by altering the composition of these materials, their attenuation capability can be enhanced [11,12,13]. 

Marble is a popular construction material around the world for the construction of buildings and external decorations. Marble is a metamorphic rock that is primarily composed of calcite (CaCO_3_) in its mineral form or dolomite (MgCO_3_), in addition to other minerals and impurities that can greatly vary. Marble is commonly used in construction due to its versatility, durability, and aesthetic appeal. Despite the benefits of marble as a material, it rapidly degrades due to its sensitivity to acid rain. Researchers are currently studying ways to alter the composition of marble to protect its integrity, but solutions are limited due to the low porosity of marble, making it difficult to add substrates to its composition. The most used solution to avoid degradation involves utilizing titania nanoparticles as a coating, which offer great photocatalytic properties that can oxidize and decompose organic and inorganic compounds that come in contact with the surface of the marble. Titania nanoparticles are applied by brush or spray directly on the surface of the marble, but because of their poor adhesion to the stone’s surface, they are easily removed by rainfall, or they penetrate the stone, reducing their effectiveness [14,15]. 

In addition to nanoparticles, researchers aim to study the radiation shielding abilities of marbles currently commercially available to determine which sample has the greatest attenuation capability. Marble has also been used as an alternative material that can be implemented as an aggregate in concrete, and the effect of increasing the amount of marble content in concrete on its neutron and gamma radiation shielding properties has been analyzed. One study found that increasing the marble content in the concrete improved the attenuation abilities of the sample [16,17]. The purpose of this study is to experimentally determine the radiation shielding ability of selected marble samples. 

## 2. Materials and Methods

### 2.1. Sampling

The samples were selected from the Egyptian market; the most available types of marble were collected. These types were produced from different quarry sites in Egypt. Ten samples of each type were collected. The samples of the same type were grouped and marked by a sample group serial number. Serial number, the commercial name of marble type, and the region of the quarry region in Egypt of each sample group are described in Table 1.

### 2.2. Sample Preparation

The samples were prepared and processed in order to perform the radiation attenuation measurements. For chemical composition determination, the samples should be in the form of homogeneous powder of the material, so a part of each sample was processed into powder and ground, and the final powder sample weight was 100 g each. On the other hand, the mass attenuation coefficient determination experiment requires samples to be solid and approximately in a regular shape to facilitate the determination of their experimental density. Their dimensions should be convenient with the detector and gamma ray source dimensions to attenuate gamma rays efficiently; the relative thin thickness of the samples is a must to achieve good geometry in the detection of the transmitted gamma rays through the sample. Thus, the samples were processed into dimensions of 5 cm length, 5 cm width, and 1 cm thickness for each sample slab. 

### 2.3. Sample Characterization

After sample preparation and processing, samples of each kind were measured to determine the average volume and weight to calculate the average density of each type of marble under investigation. The weight fractions of compounds of the materials under study were determined by using the energy dispersive spectrometer (EDS) of the analytical scanning electron microscope (JSM-5300, JEOL, JEOL Ltd., Tokyo, Japan). Samples were processed in the form of fine homogenous powders to facilitate the analysis. Three different randomly selected regions of each sample were targeted and investigated, so the overall chemical composition of the sample could be determined by averaging the chemical compositions of examining areas of the sample. The compound weight fractions of the sample represent the input of XCOM software to calculate the mass attenuation coefficient of different types of marble theoretically [18].

**Figure 1:**
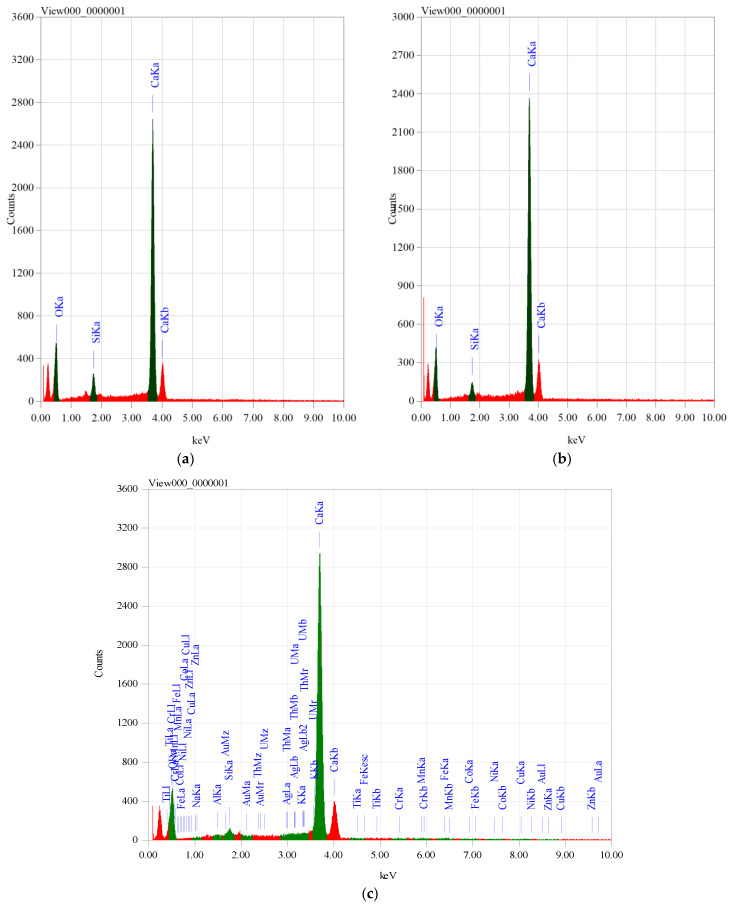
The energy dispersive spectrometer (EDS) spectra of marble: (**a**) Breshia, (**b**) Galala, (**c**) Trista.

**Figure 2:**
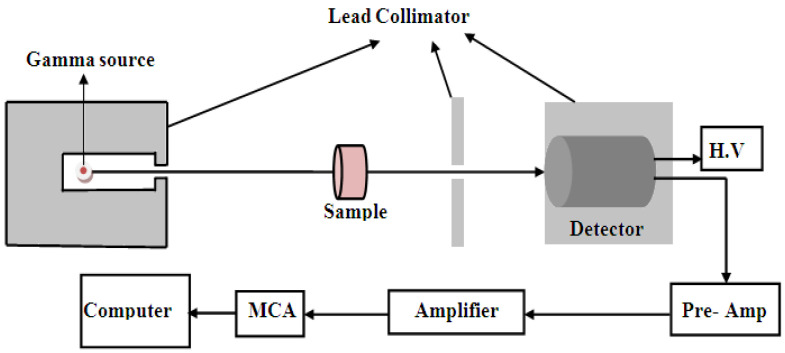
The experimental setup for the determination of the mass attenuation coefficients.

The content of calcium carbonates in marble samples was estimated from calcium oxide content by considering the equivalence between them [19]. The compositions of scanned areas of samples are illustrated in Figure 1. The average weight percent of the chemical composition of marble samples is shown in Table 2. In order to estimate the mass attenuation coefficients experimentally; we utilized the penetration of gamma rays through material, which is the basis of the gamma transmission technique [20]. The detector and the gamma source were placed at opposite sides of material on the same axis. Gamma ray intensity comes from the source calculated by the Canberra High Purity Germanium gamma ray spectrometer (HPGe), model CS20-A31CL (Radiation detection and measurements, Detroit, MI, USA), in the Institute of Graduate Studies and Research, Alexandria University, Egypt. The detector relative efficiency is 24.5% for 1333 keV of Co-60. The transmittance of gamma radiation can be estimated through narrow beam geometry. To ensure good narrow beam geometry, we used the experimental setup shown in Figure 2.

Counting time of 45 min was sufficient to obtain enough counts under each photo peak of every gamma line to achieve good statistical results with uncertainty below 1%. Ten samples of each kind of rock were investigated, and measurements were repeated five times for each to minimize errors. Then, the results of each rock type were averaged. Energy and efficiency calibration had been done before performing the attenuation measurements by using certified point sources of Am-241 (59.52 keV), Cs-137 (661.66 keV), and Co-60 (1173.23 and 1332.50 keV) to cover the desired energy range (purchased from Physikalisch-Technische Bundesanstalt PTB in Braunschweig and Berlin). The radio-specifications of used radioisotopes are given in [21,22,23,24]. The peak fitting is performed using a Gaussian shape without a low energy tail. The detected spectra of the initial and attenuated gamma photons were processed by the Genie 2000 data acquisition and analysis software made by Canberra. The mass attenuation coefficients (*μ*/*ρ*) are experimentally calculated by the following equation [25,26,27,28]:

(1) $$\mu /\rho =\frac{1}{x.\rho }ln\left(\frac{N}{N_{0}}\right)\;$$

where, *N* and *N*_0_ represent the net count rate with the presence and absence of the marble sample, respectively. The thickness of sample is denoted by (*x*).

## 3. Photon Shielding Parameters

The mass attenuation coefficient (*μ*/*ρ*) determinations via narrow beam transmission of the photon experiment and directly from the XCOM software are presented in Table 3. Additionally, the relative difference RD (in %) between the two sets of (*μ*/*ρ*) values at the investigated photon energies is shown in the table. The RD was evaluated according to the expression

**Figure 3:**
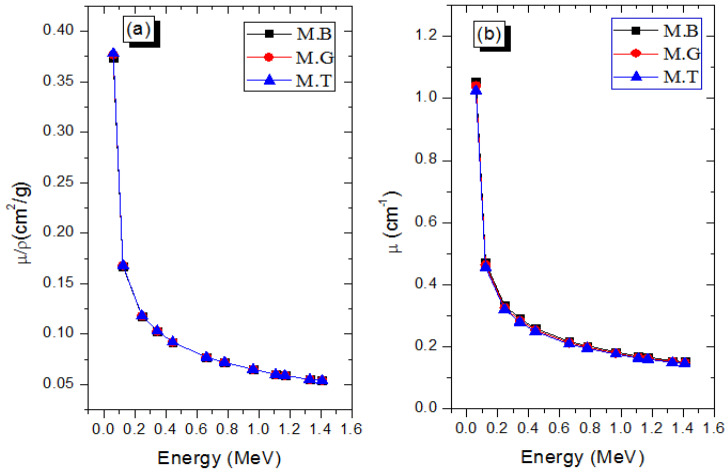
Variations of (**a**) mass and (**b**) linear attenuation coefficients with respect to photon energy of the marble samples.

(2) RD %=μρXCOM−μρExpt.μρXCOM×100

where μρXCOM and μρExpt. represent the mass attenuation coefficients obtained from the direct XCOM calculation and experimental procedure, respectively. Generally, RD varies from 0.069–0.9041% for the three marble samples and at all the considered photon energies. This clearly shows that the experimental method used a narrow beam, thin absorber, and well-collimated photon beams. Hence, the values of the μρExpt. are accurate, valid, and reliable. The variation of the photon absorption capacity with respect to the photon energy (*E*) for the marble samples was examined in terms of linear (*μ*) and mass (*μ*/*ρ*) attenuation coefficients, which are presented in Figure 3. According to the figure, both parameters decrease with *E*; the maximum μ values of 1.055, 1.041, and 1.024 cm^−1^ were obtained for M.B, M.G, and M.T, respectively, at 0.06 MeV, while the corresponding values of 0.152, 0.149, and 0.146 cm^−1^ were obtained at *E* = 1.410 MeV. The energy response of μ and (*μ*/*ρ*) is attributed to the photoelectric absorption and Compton scattering photon interaction processes. Within the energy spectrum considered, both interaction processes are significant, with CS more significant for most of the spectrum. The fact that μρ=κρ+σρ, where mass photoelectric absorption cross section (κρ∝E−3Z3) and Compton scattering cross section (σρ∝ZAE−1), explains the observed energy response of both attenuation coefficients. Furthermore, at each energy, the μρ of the marble samples are almost constant due to the similarities in the value of their mass density and chemical composition. However, the κρ∝Z3 dependence and density variation ensured a slightly noticeable difference in the μ at 60 keV, such that μ of M.B > M.G > M.T.

The effect of the chemical composition of a photon shield is always elucidated using the effective atomic number, $Z_{eff}$. $Z_{eff}$ and its variation with energy may be used to investigate the relative changes in photon absorption processes with energy for diverse shields. $Z_{eff}$ was calculated from μρ based on the equation [29,30]

(3) Zeff=∑iwiAiμρi∑iwiAiZiμρi 

**Figure 4:**
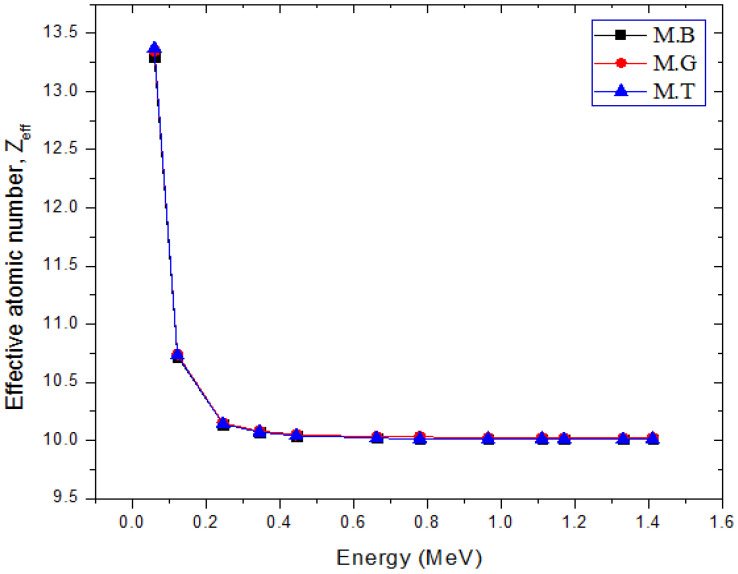
Variations of effective atomic number with respect to photon energy of the marble samples.

The identity and implication of each term in the equation have been presented previously in [29,30]. The variation of $Z_{eff}$ with *E* is presented in Figure 4. Obviously, the effective atomic number varies similarly with the attenuation coefficients. This indicates that the effective atomic number variation is guided by the partial photon absorptions κρ and σρ and their Z dependence. $Z_{eff}$ varies between 10.01 and 13.30, 10.02 and 13.35, and 10.01 and 13.37 for M.B, M.G, and M.T as *E* changes from 595 to 1410 keV. Clearly, the range of $Z_{eff}$ is within the Z of the chemical elements in the marble samples, and its value is also slightly higher for the marble that has a higher weight fraction of higher Z atom (Ca in this case) as pointed out by [30]. A strong overlapping of the $Z_{eff}$ values of the marble samples in the energy region *E* > 595 keV is due to the narrow range of value of $\frac{Z}{A}$ for the marble samples. 

**Figure 5:**
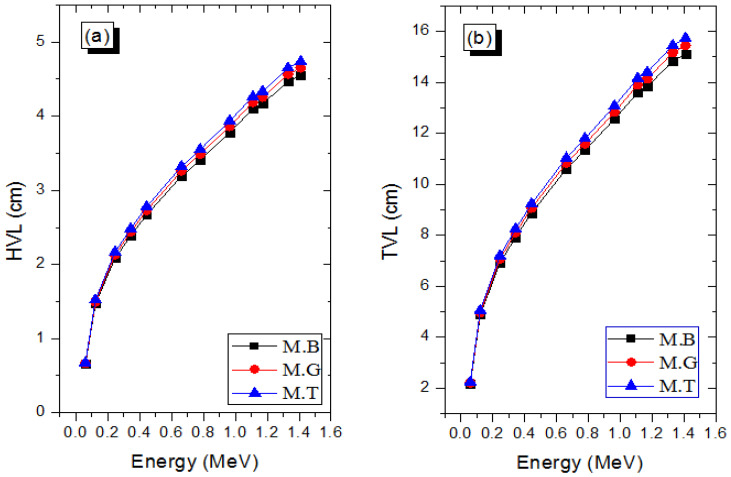
Changes in the (**a**) HVL and (**b**) TVL with E for the marbles.

The fluctuation of the half-value (HVL) and tenth-value (TVL) layer of the marble samples with E is depicted in Figure 5a and b, respectively. These two parameters are more practically used to describe the shielding ability of a material with respect to photons and also for the design of practical shields. The HVL (TVL) expresses the thickness of an absorber required to absorb only 50% (10%) of incident photon intensity in narrow beam transmission geometry. From the experimental values of μ, the HVL and TVL were approximately estimated as [31,32]: HVL=0.693μ and TVL=2.303μ.

According to Figure 5, both thicknesses increase with photon energy, an indication that photon absorption and interaction cross sections decrease as *E* rises. Hence, photons are more penetrating and required thicker materials to absorb them. The HVL (TVL) of M.B, M.G, and M.T corresponds to 4.55 (15.12), 4.65 (15.45), and 4.74 (15.73) cm at the peak energy considered herein (1.410 MeV). Based on these values, it is clear that the photon absorption competence of the marble samples is in the order M.B > M.G > M.T; this is consistent with the trend of the marbles samples’ mass densities.

**Figure 6:**
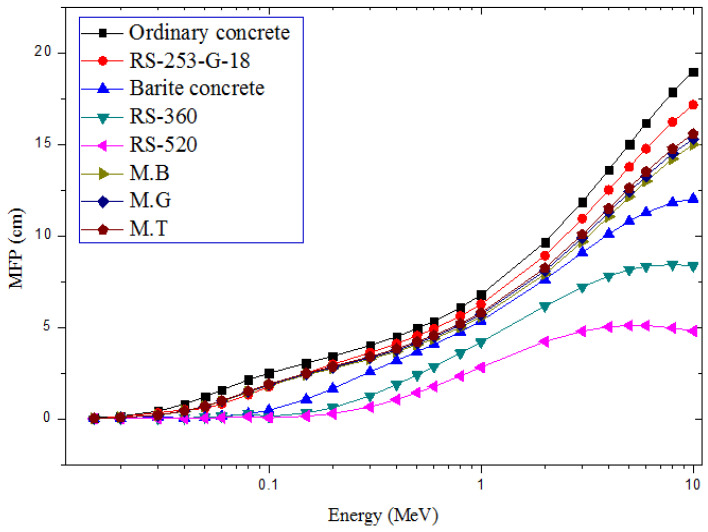
Comparison of MFP of the marble samples with orthodox photon shields.

The narrow beam shielding ability of the present marble samples is compared with those of ordinary concrete [33], barite concrete [33], RS-253-G18, RS-360, and RS-520 shielding glasses [34] for photon energies between 0.15 and 10 MeV, as presented in Figure 6. The MFP ($MFP=\mu^{-1}$) is the average distance between photon interaction within the marble shield. A higher value of MFP is associated with inferior shielding competence. Consequently, Figure 4 shows that the three marble samples have better shielding abilities compared to ordinary concrete and RS-253-G18 commercial glass shields. Hence, the marble samples could be adopted for structural shielding instead of RS-253-G18 in applications where optical transparency of the shield is not a major requirement. Furthermore, rather than using ordinary concrete of a high thickness, these marbles stones could be adopted for laminating existing concrete walls when facilities are to be upgraded or when space is a constraint. This will reduce the amount (thickness) of concrete shield, reduce the cost of using pure marble for the shielding purpose, and also provide efficient radiation attenuation. These types of marble could thus be used to replace or compliment these conventional shields depending on the basic shielding features required in nuclear facilities where photons are used.

**Figure 7:**
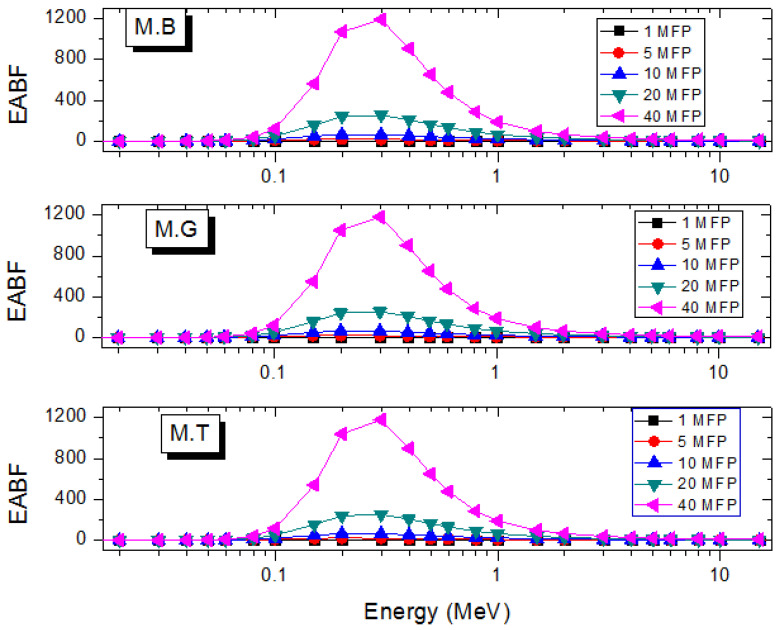
Variation of EABF of the marble samples with energy at selected depths.

**Figure 8:**
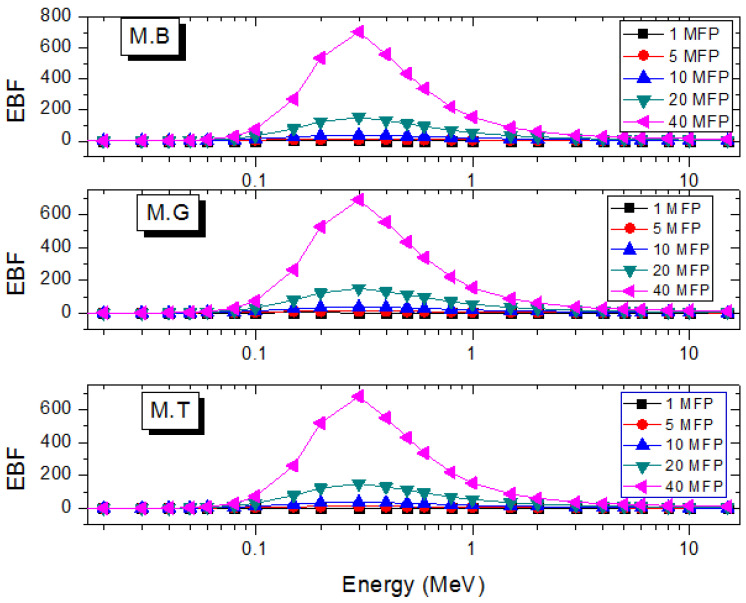
Variation of EBF of the marble samples with energy at selected depths.

**Figure 9:**
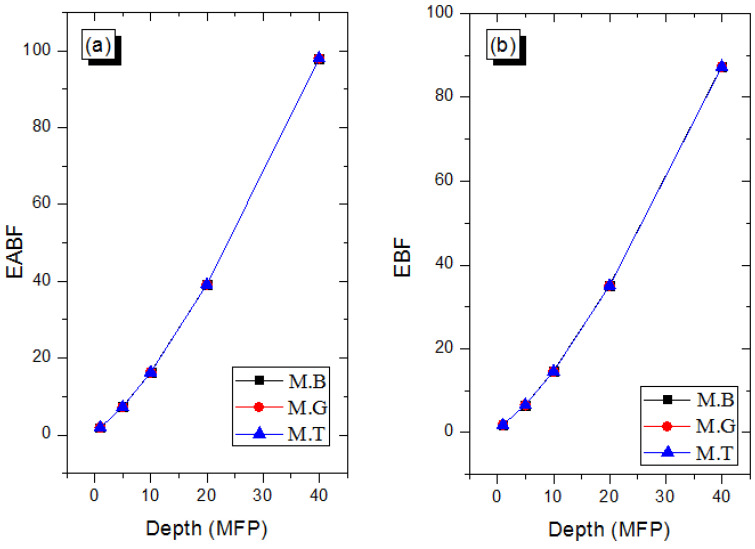
Variation of EABF (**a**) and EBF (**b**) with depth in the marble samples *E* = 1.5 MeV.

In many practical applications of photons where shields are required, the narrow beam transmission becomes a scarce luxury. The estimation of shielding parameters in the narrow beam thus requires corrections to account for the creation of scattered and secondary photons that are transmitted through the shields. The photon buildup factor *B* serves this correction purpose [35,36]. The exposure (EBF) and energy absorption (EABF) buildup factors are two kinds of *B* that are often estimated for potential shields [36]. Figure 7 and Figure 8 depict the EABF and EBF as functions of E at selected depths in the marble samples. *B* is generally high in the energy region where Compton scattering dominates proceedings due to multiple photon scattering. On either side of the *B* peak, total photon absorption through the photoelectron and pair creations respectively lead to diminished EABF and EBF. As shown in Figure 9a,b, EABF and EBF increase with depth as a result of multiple scattering as marble thickness rises at a photon energy of 1.5 MeV. Furthermore, all three marble types scatter photons similarly, hence the almost constant EABF and EBF. Clearly, there is not much difference in the photon scattering and absorption abilities of the marble samples. However, they can perform better than existing shields in terms of photon absorption prowess.

**Figure 10:**
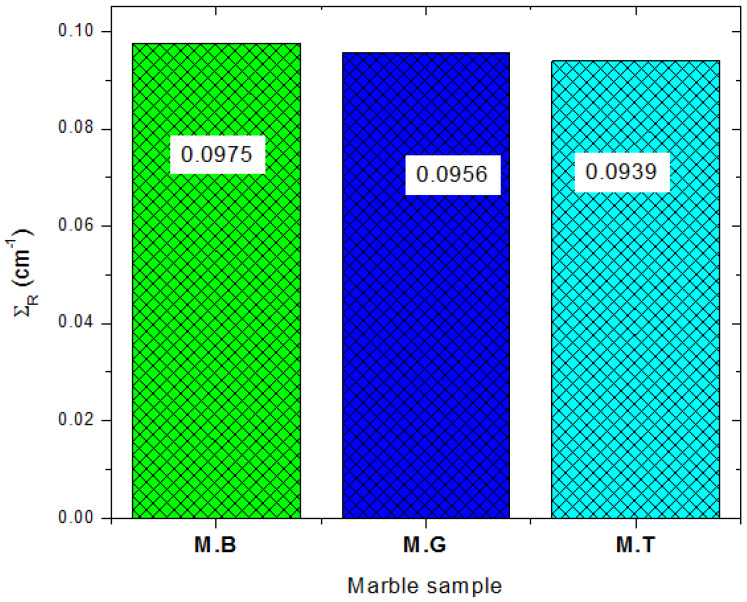
Effective removal for fast neutrons of the marble samples.

The fast neutron removal cross section $\Sigma_{R}$ expresses the ability of the marbles to slow down fast neutrons through multiple scattering. It is the ability of the marbles to shield fast neutrons. The $\Sigma_{R}\;$ of the marbles, as shown in Figure 10, has value of 0.0975, 0.0956, and 0.0939 cm^−1^ for MB, MG, and MT, respectively. The trend of the $\Sigma_{R}$ is consistent with the density of the marble samples and also the partial density of the O atom in the samples. The partial density value for MB, Mg, and MT is 1.3601, 1.3259, and 1.2992 g/cm^3^, respectively. Compared to ordinary concrete with $\Sigma_{R}=0.0937$ cm^−1^, the investigated marble samples have a better fast neutron shielding ability.

## 4. Conclusions

This study calculated the mass attenuation coefficient of some marble samples found in Egypt experimentally and theoretically by XCOM, and a good agreement was found between the two methods. The HVL (TVL) of M.B, M.G, and M.T corresponds to 4.55 (15.12), 4.65 (15.45), and 4.74 (15.73) cm at the peak energy considered herein (1.410 MeV). Based on these values, it is clear that the photon absorption competence of the marble samples is in the order M.B > M.G > M.T. The *Z_eff_* was also determined, and it was found to have the same values for the three samples with different energies. The MFP was calculated and compared with some shielding materials and showed its superiority over some of them. The exposure and energy absorption buildup factor were calculated for the samples at different mfp to show the extent of the absorption of gamma rays. The $\Sigma_{R}$ was calculated and compared to concrete and showed that the investigated marbles have a better fast neutron shielding ability. The implication of this is that for nuclear reactor shields and in high energy photon applications where the possibility of the reaction (γ, n) is high, the use of the present marbles as laminators of concrete shields will help to absorb the produced neutrons better than ordinary concrete.

## Figures and Tables

**Table 1:** The samples, marking, and description.

S.N	Commercial Name of Marble Samples	Production Region
M.B	Breshia	Sinai
M.G	Galala	Suez
M.T	Trista	Sinai

**Table 2:** Chemical composition of the marble samples.

Sample	Chemical Composition (Weight %)					Density (g·cm^−3^)
	Na_2_O	Al_2_O_3_	SiO_2_	K_2_O	CaCO_3_	
M.B	‒	‒	5.24	‒	94.76	2.82
M.G	‒	‒	3.52	0.325	96.155	2.76
M.T	0.2	0.36	1.52	0.16	97.76	2.71

**Table 3:** Relative differences between mass attenuation coefficient from experimental and XCOM calculations.

Energy (MeV)	M.B			M.G			M.T		
	XCOM	Expt.	R.D (%)	XCOM	Expt.	R.D (%)	XCOM	Expt.	R.D. (%)
0.060	0.375	0.374	0.216	0.377	0.377	0.1142	0.379	0.378	0.2453
0.122	0.167	0.167	0.0802	0.167	0.168	0.3371	0.168	0.168	0.2378
0.245	0.118	0.118	0.1931	0.118	0.118	0.2232	0.118	0.118	0.2355
0.344	0.103	0.103	0.2628	0.103	0.103	0.2519	0.103	0.103	0.2505
0.444	0.092	0.092	0.3491	0.092	0.092	0.3529	0.092	0.092	0.3505
0.662	0.077	0.077	0.6154	0.077	0.077	0.6139	0.077	0.077	0.6084
0.779	0.072	0.072	0.1689	0.072	0.072	0.1718	0.072	0.072	0.178
0.964	0.065	0.065	0.1267	0.065	0.065	0.1307	0.065	0.065	0.1374
1.110	0.061	0.06	0.9041	0.061	0.06	0.900	0.061	0.06	0.8931
1.170	0.059	0.059	0.069	0.059	0.059	0.0733	0.059	0.059	0.0803
1.330	0.055	0.055	0.4098	0.055	0.055	0.4055	0.055	0.055	0.3984
1.410	0.054	0.054	0.7507	0.054	0.054	0.7548	0.054	0.054	0.7618

## Data Availability

The data presented in this study are available on request from the corresponding author.
